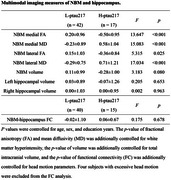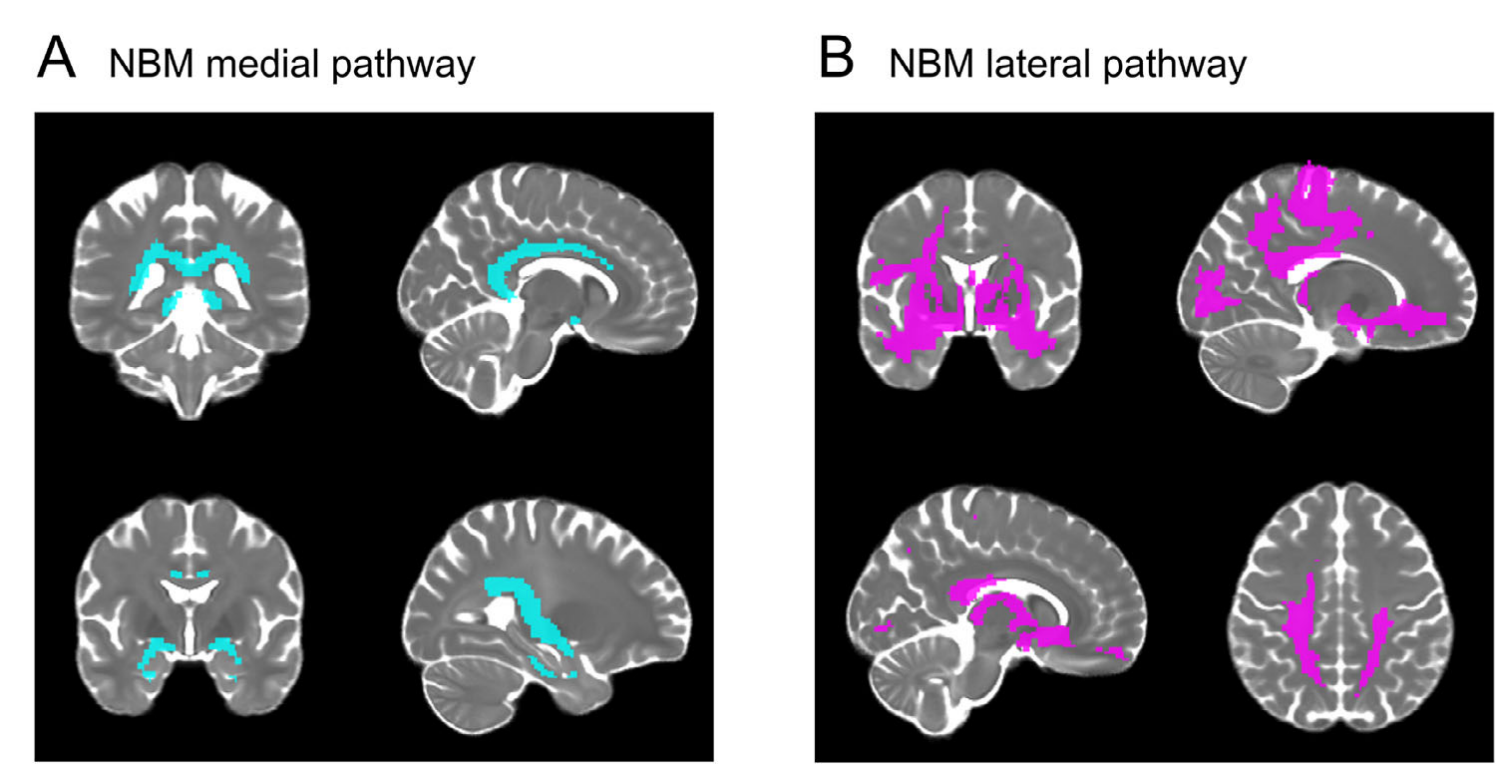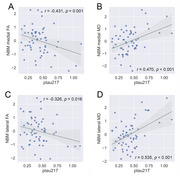# Cholinergic White Matter Degeneration Could Help Identify Cognitively Unimpaired Elderly With Higher Plasma Ptau217

**DOI:** 10.1002/alz70856_097010

**Published:** 2025-12-24

**Authors:** Qian Chen, Bing Zhang

**Affiliations:** ^1^ Department of Radiology, Nanjing Drum Tower Hospital, Affiliated Hospital of Medical School, Nanjing University, Nanjing, Jiangsu, China; ^2^ Nanjing Drum Tower Hospital, Nanjing, Jiangsu, China

## Abstract

**Background:**

The nucleus basalis of Meynert (NBM), a key nucleus that projects cholinergic neurons to the cerebral cortex, is one of the earliest regions affected in Alzheimer's disease (AD), preceding changes in medial temporal structures. However, the associations between NBM multimodal imaging markers and the newly proposed AD Core 1 pathological biomarker of plasma phosphorylated tau 217 (ptau217) remain unclear.

**Method:**

In this study, fifty‐nine cognitively unimpaired elderly individuals were enrolled. Based on whether an individual's plasma ptau217 value was higher or lower than the group mean, the participants were divided into two groups: the higher ptau217 group (H‐ptau217, n = 17) and the lower ptau217 group (L‐ptau217, n = 42). NBM volume, cholinergic white matter pathway integrity, and its functional connectivity with the hippocampus were calculated. Correlation, mediation, and receiver operating characteristic (ROC) curve analyses were performed to investigate the relationships between plasma ptau217 and NBM multimodal imaging markers.

**Result:**

There were no significant differences in demographic, psychological, and cognitive measures between the L‐ptau217 group and H‐ptau217 groups. Compared to the L‐ptau217 group, the H‐ptau217 group showed decreased fractional anisotropy and increased mean diffusivity in both the medial and lateral cholinergic white matter pathways. No significant differences were observed between the groups in NBM and hippocampal volumes or in functional connectivity. Higher plasma ptau217 were associated with lower fractional anisotropy and higher mean diffusivity in both the medial and lateral cholinergic white matter tracts. Furthermore, cholinergic white matter measures significantly mediated the relationship between plasma ptau217 and memory and executive function. The ROC curve based on cholinergic white matter measures showed an area under the curve of 0.906 in distinguishing between L‐ptau217 group and H‐ptau217 groups.

**Conclusion:**

Our study extends previous findings by highlighting the potential of MRI‐derived measures of NBM cholinergic white matter degeneration to capture the initial pathological events of elevated plasma ptau217. This may provide insights into the role of NBM cholinergic white matter degeneration in the preclinical identification of potential AD patients with Core 1 biomarker of plasma ptau217 before the occurrence of objective cognitive impairment.